# Chemical Constituents of *Salix babylonica* L. and Their Antibacterial Activity Against Gram-Positive and Gram-Negative Animal Bacteria

**DOI:** 10.3390/molecules24162992

**Published:** 2019-08-18

**Authors:** Eddy Nathalye González-Alamilla, Manases Gonzalez-Cortazar, Benjamín Valladares-Carranza, Marco Antonio Rivas-Jacobo, Camelia Alejandra Herrera-Corredor, Deyanira Ojeda-Ramírez, Adrian Zaragoza-Bastida, Nallely Rivero-Perez

**Affiliations:** 1 Facultad de Agronomía y Veterinaria, Universidad Autónoma de San Luis Potosí, Carretera San Luis-Matehuala Km, 14.5, Ejido Palma de la Cruz, 78321 Soledad de Graciano Sánchez, San Luis Potosí, Mexico; 2 Centro de Investigación Biomédica del Sur, Instituto Mexicano del Seguro Social, Argentina No. 1. Col. Centro, CP 62790 Xochitepec, Morelos, Mexico; 3 Facultad de Medicina Veterinaria y Zootecnia Universidad Autónoma del Estado de México, El Cerrillo Piedras Blancas, C.P. 50295 Toluca, Estado de México, Mexico; 4 Área Académica de Medicina Veterinaria y Zootecnia, Instituto de Ciencias Agropecuaria, Universidad Autónoma del Estado de Hidalgo, Av. Universidad Km 1, Ex-Hda. de Aquetzalpa, 43600 Tulancingo, Hgo, Mexico

**Keywords:** *Salix babylonica* L. hydroalcoholic extract, luteolin, luteoloside, antibacterial activity

## Abstract

The principle of animal wellbeing, which states that animals should be free from pain, injury, and disease, is difficult to maintain, because microorganisms are most frequently found to be resistant or multi-resistant to drugs. The secondary metabolites of plants are an alternative for the treatment of these microorganisms. The aim of this work was to determine the antibacterial effect of *Salix babylonica* L. hydroalcoholic extract (SBHE) against *Escherichia coli, Staphylococcus aureus* and *Listeria monocytogenes*, and identify the compounds associated with the activity. The SBHE showed activity against the three strains, and was subjected to a bipartition, obtaining aqueous fraction (ASB) with moderate activity and organic fraction (ACSB) with good activity against the three strains. The chromatographic separation of ACSB, allowed us to obtain ten fractions (F1AC to F10AC), and only three showed activity (F7AC, F8AC and F10AC). In F7AC, five compounds were identified preliminary by GC-MS, in F8AC and F10AC were identified luteolin (1) and luteolin 7-*O*-glucoside (2) by HPLC, respectively. The best antibacterial activity was obtained with F7AC (*Listeria monocytogenes*; MIC: 0.78 mg/mL, MBC: 0.78 mg/mL) and F8AC (*Staphylococcus aureus;* MIC: 0.39 mg/mL; MBC: 0.78 mg/mL). The results indicated that the compounds obtained from SBHE can be used as an alternative treatment against these microorganisms and, by this mechanism, contribute to animal and human health.

## 1. Introduction

According to the Terrestrial Animal Health Code of World Organization of Animal Health (OIE), animal wellbeing is defined as the physical and mental state of an animal in relation to the conditions in which it lives and dies and its relationship with five freedoms: 1. Freedom of thirst, hunger, and malnutrition; 2. Freedom from discomfort and exposure; 3. Freedom from pain, injury, and disease; 4. Freedom from fear and distress; and 5. Freedom to express normal behavior [1,2]. Concerning the third freedom, in 1994, Webster stated that animal diseases should be treated rapidly, although bacterial infections that once were easily treated are becoming untreatable [3,4].

The introduction of antibiotics into clinical practice represented one of the most important interventions for the control of infectious diseases. However, antibiotics overuse has deteriorated the effectiveness of these drugs, meaning that infectious diseases will be more difficult to treat [5]. Due to the lack of effective therapies, the discovery of new antibiotics is a challenge in animal and human health, and plants are an important alternative as new antimicrobial drugs [6,7,8,9,10].

In animals, there are different biological agents that can cause dangerous diseases because of their deleterious effects on animal welfare and their zoonotic potential. Such is the case for *Escherichia coli,* a bacterial commensal of the intestinal microflora of a variety of animals, including humans. *Escherichia coli* cause diseases in mammals and birds. The Pathogenic *Escherichia coli* strains fall into two categories: those that cause intestinal pathologies and those that cause extraintestinal pathologies. Intestinal pathologies mostly consist of severe diarrhea, potentially evolving into a hemolytic uremic syndrome, a pathology that can lead to death [11].

On the other hand, *Staphylococcus aureus* is one of the most important pathogens in veterinary medicine. Among the susceptible species of infection for this bacterium are bovine, sheep, goat, pig, horse, cat, dog, poultry and human. The main infections that are reported in the species mentioned above are mastitis, impetigo, dermatitis, mild folliculitis, mammary botryomycosis, castration wounds, urinary tract infection and abscesses [12].

Listeriosis, caused by *Listeria monocytogenes,* is a disease of, birds, fish, crustaceans and humans. Listeriosis is characterized by septicemia, encephalitis, meningitis, meningoencephalitis, abortion, stillbirth, perinatal infections, and gastroenteritis [13,14].

*Salix babylonica,* commonly known as weeping willow, belongs to the genus, *Salix,* of the family, Salicaceae, is the most known species of the willows, distributed in some areas of Asia, Europe, and America. It has been used as an ornamental and medicinal plant and is considered an important tree for the study of its phytochemical properties [7].

The biological properties associated with *Salix babylonica* are anthelmintic, antiseptic, antiarthritic, astringent, analgesic, anticarcinogenic, antipyretic, antimalarial, antioxidant, antifungal, anthelmintic, and antibacterial, and these activities are related to the content of secondary compounds, such as the benzyl ester of gentisic acid 2′-*O*-acetyl β-d-glucoside, trichocarpin, salicin, kaempferol 7-*O*-glucoside, apigenin 7 *O*-galactoside, luteolin 4′-*O*-glucoside, ester of terephthalic acid, tritetracontane, octadecenoic acid-1,2,3-propanetriyl ester, hexadecenoic acid-methyl ester, and 1,3-dioxane-4-(hexadecyloxy)-2-pentadecyl [7,15,16].

Considering this information, the aim of this work was to determine the antibacterial effect of *Salix babylonica* L. hydroalcoholic extract (SBHE) against *Escherichia coli, Staphylococcus aureus* and *Listeria monocytogenes*, and identify the compounds associated with the activity.

## 2. Results

### 2.1. Minimal Inhibitory Concentration (MIC)

The broth microdilution method was used to determine the Minimal Inhibitory Concentration for hydroalcoholic extract, fractions, and subfractions of *Salix babylonica.* Table 1 shows that SBHE inhibits the growth of Gram-positive and Gram-negative bacteria and that ACSB was more active than ASB. For this reason, only ACSB was fractioned, obtaining F1AC to F10AC subfractions. These subfractions were evaluated in the antibacterial test. Only F7AC, F8AC, and F10AC showing antibacterial activity, F7AC was the most active treatment against *Listeria monocytogenes* (MIC = 0.78 mg/mL, *p* = 0.0001). In contrast, F8AC was statistically (*p* = 0.0001) more active than F7AC against *Staphylococcus aureus* (MIC = 0.39 and 1.56 mg/mL, respectively). Finally, Gram-negative bacteria *Escherichia coli* was more sensitive to ACSB and F7AC, showing an MIC of 6.25 mg/mL for both. These results were statistically lower (*p* = 0.0001) than the MIC values, obtained for SBHE, ASB, and F8AC, for this bacterium.

### 2.2. Minimum Bactericidal Concentration (MBC)

The Minimum Bactericidal Concentration (MBC) was determined by the lowest concentration of the compound, at which no colony-forming units (CFUs) were detected on solid media. The MBC values corresponding to the extract and fractions were isolated, as shown in Table 2. SBHE induced the death of *Staphylococcus aureus* (50 mg/mL), *Listeria monocytogenes* (100 mg/mL) and *Escherichia coli* (200 mg/mL), and the aqueous fraction (ASB) only has activity in *Listeria monocytogenes* (100 mg/mL), while the organic fraction (ACSB) has antibacterial activity in the three microorganisms challenged, *Listeria monocytogenes*, *Staphylococcus aureus* and *Escherichia coli* (3.12, 12.5, and 50 mg/mL). F7AC was the most effective fraction (*p* = 0.0001) and killed the 99.9% of *Listeria monocytogenes* at 0.78 mg/mL, but it showed no effect on *Escherichia coli*. On the other hand, F8AC showed the best bactericidal activity against *Staphylococcus aureus* at 0.78 mg/mL (*p* = 0.0001) and on *Listeria monocytogenes* and *Escherichia coli* (1.56 and 25 mg/mL, respectively). F10AC induced the death of the three microorganisms, but its concentration was higher (25 mg/mL) than the F7AC and F8AC concentration.

### 2.3. Identification of Major Compounds

The hydroalcoholic extract (SBHE) exhibits an important antibacterial activity (Table 1 and Table 2), a liquid partition separation was performed to obtain two fractions: aqueous (ASB) and organic (ACSB), afterwards the active fraction ACSB (Table 1 and Table 2) was purified in a normal phase column chromatographic obtaining 10 subfractions (F1AC to F10AC). Due to only F7AC, F8AC and F10AC showed antibacterial effect (Table 1 and Table 2), these fractions were analyzed by HPLC and GC-MS in order to identify their chemical composition.

**Figure 1:**
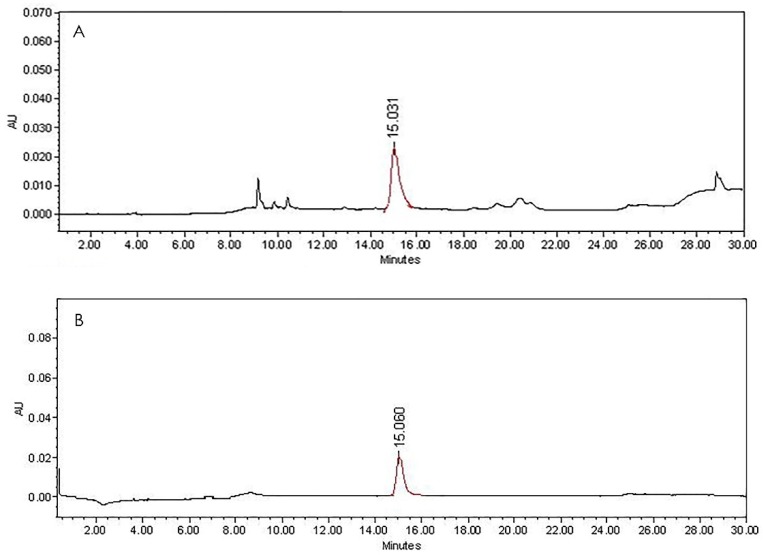
HPLC chromatogram of the (**A**) F8AC fraction and (**B**) Luteolin analytical standard.

**Figure 2:**
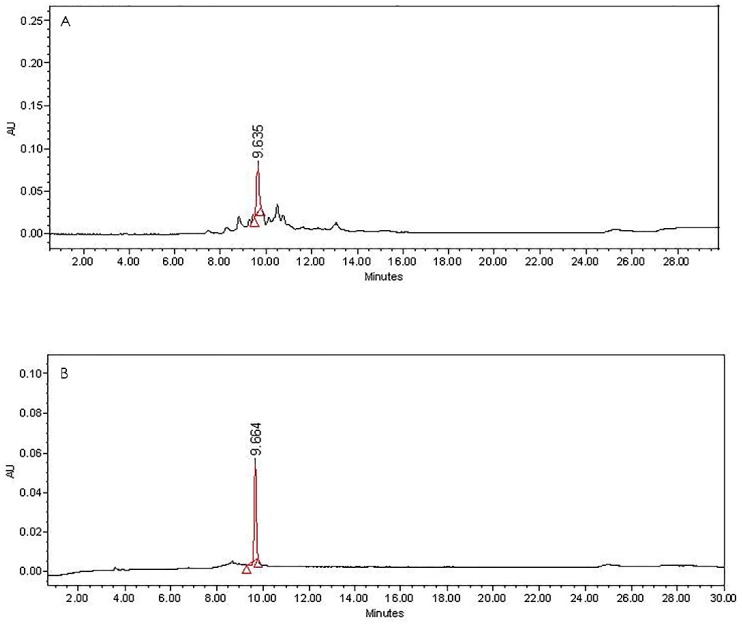
HPLC chromatogram of the (**A**) F10AC fraction and (**B**) Luteoloside analytical standard.

**Figure 3:**
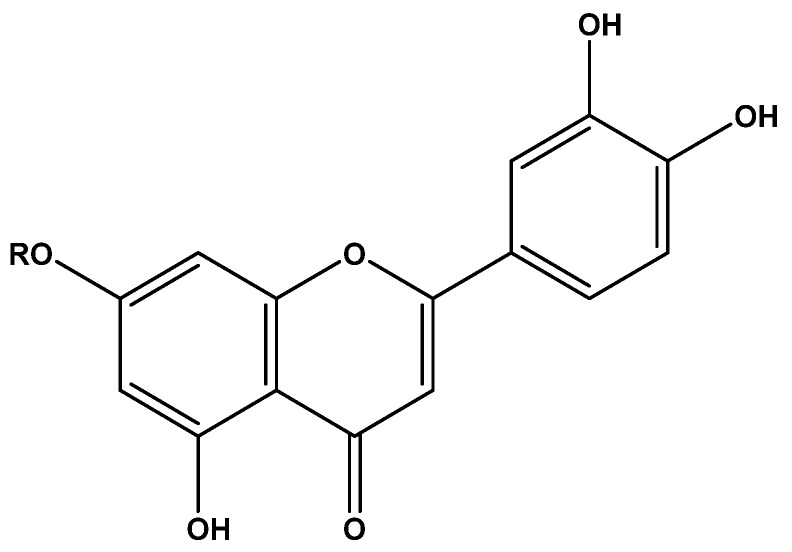
Chemical structure of 3′,4′,5,7-tetrahydroxyflavone or luteolin (**1**) and 3′,4′,5,7-tetrahydroxyflavone 7-*O*-glucoside or luteoloside (**2**). (**1**) R = H; (**2**) R = Glucose.

F8AC and F10AC were subjected to an HPLC method which revealed that these fractions contain the flavonoids: luteolin (1) and luteoloside (2), respectively. These flavones were identified by direct comparison by HPLC with analytical standards (Figure 1 and Figure 2). The chemical structure of these compounds can be shown in the Figure 3.

**Figure 4:**
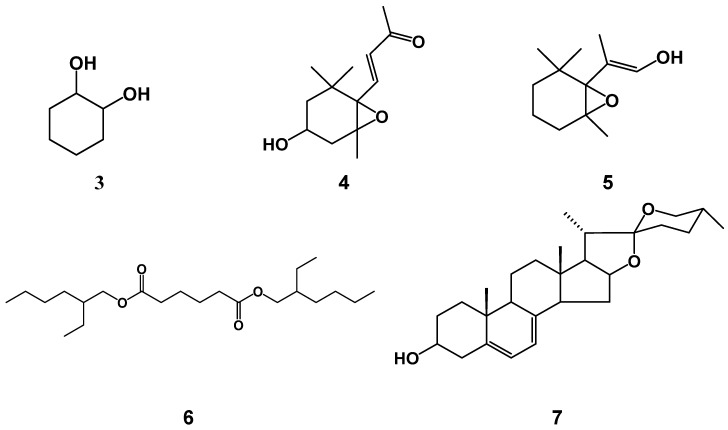
Chemical structure of compounds (**3**–**7**), identified preliminary by GC-MS from F7AC fraction.

On the other hand, the analysis of F7AC by GC-MS suggests preliminary that this fraction is a mixture of five compounds: three alcohols (**3**–**5**) an ester (**6**) and a sapogenin (**7**), which are listed according their elution order in the Table 3. The preliminary identification of these compounds allowed propose their chemical structures that are showing in Figure 4. However, more purification must be made in order to obtain the pure compounds and identify them unequivocally through the analysis of their NMR ^1^H and ^13^C data.

## 3. Discussion

Antibiotics overuse has deteriorated the effectiveness of these drugs on some microorganism, so that, new antibacterial agents is required, plant-derived extracts and compounds are an alternative source of antimicrobial agents because of their low toxicity in human cells and limited effects on the environment [10,17].

The presents study shows that the hydroalcoholic extract of *Salix babylonica* (SBHE), and its fractions ASB and ACSB possess activity against the tested bacteria (Table 1 and Table 2). The MIC obtained with SBHE was higher (25 to 50 mg/mL) than that obtained with the ASB (3.12 to 25 mg/mL) and ACSB fractions (1.56 to 6.25 mg/mL). These results indicate that the bipartition increased the antibacterial activity, and this effect can explain the possible antagonism between compounds within the extract, which are separated by their polarity and increased in terms of their biological activity [18]. 

There are studies in which the antibacterial activity of members of *Salix* genus have been evaluated. In 2013, Sulaiman et al. conducted a study, where evaluated the antibacterial activity of *Salix alba* ethanolic extract from bark, and found that *Salix alba* had good activity against *Staphylococcus aureus*, medium activity against *Pseudomonas aeruginosa* and no possesses effect against *Escherichia coli* and *Klebsiella pneumoniae*. The concentrations used were 10, 20, 40, 60, and 80 mg/mL, using the agar diffusion technique, and the highest inhibition zone (IZ) was obtained for *Staphylococcus aureus* at 80 mg/mL. The effect showed for this extract is similar to the observed in our experiment for SBHE, since both extracts had a better effect against Gram-positive bacteria (*Staphylococcus aureus* and *Listeria monocytogenes*) than Gram-negative bacteria (*Escherichia coli*). [14].

Popova et al., in 2015 evaluated the antibacterial effect of a *Salix babylonica* aqueous extract of leaves and seeds, and obtained MIC values of 56 mg/mL for *Escherichia coli*, 60 mg/mL for *Staphylococcus aureus*, 62 mg/mL for *Salmonella enterica,* and 104 mg/mL against *Paenibacillus alvei* [19]. Our MIC values obtained for hydroalcoholic extract SBHA are highest against *Escherichia coli* (100 mg/mL) and lower for *Staphylococcus aureus* (25 mg/mL), but we only used the leaves of the plant and the extraction technique was different, we use maceration and they use decoction. 

In addition, Wahab et al. (2018) determined that methanolic extracts of leaves and bark of *S babylonica* possesses antibacterial activity against *Pseudomona aeruginosa* (10 mm, IZ), *Klebsiella pneumoniae* (9 mm, IZ), *Escherichia coli* and *Staphylococcus aureus* (8 mm, IZ) at 100 µg/mL. However, these results are not comparable with those observed in the present experiment, because the antibacterial assay and the concentrations evaluated were different. Moreover, Wahab et al. used dimethyl sulfoxide (DMSO) to dilute the extracts [7], and this compound increases the permeability of the bacterial membrane, increasing the activity of the compounds and reducing the concentrations of use [20]. Despite, there are some studies that demonstrated the antibacterial capacity of *Salix babylonica* extracts, the active compounds remained unknowing until now.

Regarding it, the best MIC values were obtained for the organic fraction (ACSB) (Table 1). These results suggested the presence of secondary metabolites in ACBS capable of inhibited and killed these bacteria. For this reason, we continued with a bioguided chemical fractionation in order to identify the active antibacterial compounds in ACSB. All fractions obtained from ACSB fractionation were submitted to the MIC pharmacological test. As illustrate Table 1, only F7AC, F8AC, and F10AC showed antibacterial activity, and they were most active against Gram-positive bacteria *Staphylococcus aureus* and *Listeria monocytogenes*. 

This effect can be explained due to Gram-negative bacteria structure, because these microorganisms have a double phospholipid membrane that protects the cell wall from lipophilic solutes, and the porins constitute a selective barrier for hydrophilic solutes, so the bacteria is protected from being penetrated by compounds, such as antibiotics or some secondary metabolites, derived from plants [10,21,22].

The HPLC analysis of F8AC and F10AC showed the presence of two flavonoids: 3′,4′,5,7-tetrahydroxyflavone, namely luteolin (**1**); and 3′,4′,5,7-Tetrahydroxyflavone-7-*O*-glucoside (**2**) namely luteoloside, respectively.

Luteolin is a flavone present in fruits, vegetables, and medicinal herbs, this compound has a C6–C3–C6 structure, possessing two benzene rings, a third, oxygen-containing (C) ring, and a 2−3 carbon double bond. It also possesses hydroxyl groups at carbons 5, 7, 3′, and 4′ positions, and the biological and biochemical properties of this flavonoid are associated with the hydroxyl groups and the 2−3 double bond, and luteoloside is the natural form in which luteolin is found in plants. Its structure is changed when it is hydrolyzed to free luteolin during absorption in the gut, and it is only detected in plasma and urine as sulfate or glucuronide phase II-conjugates [23,24,25].

There are some reports about the antibacterial properties of flavones lutelin (1) and luteoloside (**2**). For example, a fraction obtained from a methanolic extract of aerial parts of *Pedicularis wilhelmsiana* that contained lutein and luteoloside was active against the Gram-positive bacteria *S. epidermidis* (MIC = 23 mg/mL), *M. luteus* (MIC = 26.9 mg/mL) and *Staphylococcus aureus* (Inhibition zone (IZ) = 14.1 at 100 mg/mL), and the Gram-negative bacteria *Psudomona aureginosa* (IZ = 14.4 mm at 100 mg/mL) [26]. In addition, Tian et al. (2018) evaluate the antibacterial capacity of a rich flavonoid fraction obtained from leaves of *Abutilon theophrasti* Medic, which show MIC values of 1.02, 0.51, 0.06 and 0.26 g/mL against *Escherichia coli*, *Salmonella* spp., *Staphylococcus aureus* and *Streptococcus* spp., respectively. The HPLC-DAD-ESI-MS analysis of this fraction develop the presence of rutin, quercetin 7-*O*-β-glucoside, kaempferol 3-*O*-α-rhamnopyranosyl(1→6)-β-glucopyranoside, luteolin, apigenin 7-*O*-β-diglucoside, poncirin, and tiliroside [27].

Recently, Khazaeli et al. (2019) evaluated the antibacterial activity of a henna oil which contained 56.57 ± 0.66 μg/mL of luteolin, and found that was active against *Gardnerella vaginalis* (CMI = 87 μg/mL, CMB = 870 μg/mL), *Neisseria gonorrhoeae* (CMI = 87 μg/mL, CMB = 870 μg/mL) and Streptococcus (CMI = 870 μg/mL, CMB = 8700 μg/mL) [28]. Furthermore, luteoloside isolated from *Lonicera japonica* leaves had bacteriostatic effect against *Escherichia coli* and *Staphylococcus aureus* with inhibition zones of 22.9 and 27.4 mm, respectively, at a concentration of 2 mg/disc [29], and Wu et al. (2013) determine the MIC_50_ (concentration that inhibits the growth of 50% of organisms) of luteolin against *Escherichia coli* (MIC_50_ = 67.25 µg/mL) [30]. In the present experiment, luteolin exhibited a MIC and MCB of 25 mg/mL against the same bacteria using broth microdilution method, although these concentrations are highest, we demonstrated that luteolin has a bactericidal effect [10].

On the other hand, the major component identified preliminary by GC-MS in F7AC was the (*E*)-2-(2,2,6-trimethyl-7-oxabicyclo [4.1.0]heptan-1-yl) prop-1-en-1-ol (**5**), an epoxy β-ionone derivative. β-ionone is a cyclic terpenoind, it and some of their derivatives occur in plants, fruits, vegetables and grains that containing β-carotene. Previous studies have demonstrated that these compounds can exhibit significant pharmacological activities such as antileishmanial, anti-inflammatory, antifungal and antibacterial activities [31]. Chunsriimyatav et al (2013) identify three β-ionone derivatives with similar structure to **4** and **5,** from *Polygonatum odoratum* leaves: 4-(1,3,3-trimethyl-7-oxabicyclo[4.1.0]hept-2-yl)-2-pentanone,2-methyl-4-(1,3,3-trimethyl-7-oxabicyclo[4.1.0]hept-2-yl)-3-Buten-2-ol and 2-(2,2,6-trimethyl-7-oxabicyclo[4.1.0]hept-1-yl)-propenyl ester of acetic acid [32]. 

However, there are no other reports about the pharmacological properties of this kind of oxabiciclic compounds. In 2012, Sharma et al evaluated the antibacterial activity of 22 β-ionones derived chalcones against *Bacillus subtillus*, *Staphylococcus aureus*, *Escherichia coli*, *Pseudomona aureginosa*, methecillin resistant *Staphylococcus aureus* and *Salmonella typhi* and found MIC values of 3.12 to >100 µg/mL for all the bacteria except for *Staphylococcus aureus*, in these cases, the values were from 4 to 20 mg/mL [33]. The difference observed in the antibacterial activity between these compounds and our β-ionone derivatives could be due to presence of epoxy group in compounds **4** and **5**. However, further studies are necessary to establish it, and as we mentioned previously, it is necessary to submit the F7AC fraction to further chromatographic separations in order to obtain the spectrometric data of pure compounds that confirm the presence of compounds **4** and **5**, as well as evaluate the antibacterial capacity of them to identify the active compound in this fraction.

On the other side, the MBC is an important parameter that allows determinate the capacity of some compounds to kill microorganisms and can be used to determine if the compounds have bacteriostatic or bactericidal power, when the ratio of MBC/MIC is calculated [5,10]. Djihane et al. (2017) found that the effect is bacteriostatic, when the ratio is greater than 4, and bactericidal, when the ratio is less than or equal to 4 [10]. According our results, the flavones luteolin and luteoloside isolated from *Salix balylonica* possess bactericidal power, because ratio MBC/MIC were between 0.5 and 4. This result is important, because it confirms that the compounds isolated not only inhibit the bacterial growth (MIC), but also kill 99.9% of the initial bacterial population (MBC) [5,10,34,35].

In conclusion, the results of the present study indicated that the compounds isolated from *Salix babylonica* hydroalcoholic extract could be a natural and functional alternative for treatment diseases caused by *Escherichia coli, Staphylococcus aureus,* and *Listeria monocytogenes.*

## 4. Materials and Methods

### 4.1. Plant Material

The leaves of *Salix babylonica* were harvested from Tulancingo de Bravo, located in the State of Hidalgo, Mexico (20°05′09″ N 98°21′48″ W), in the June–August period. For plant identification, the Herbarium of UNAM (Universidad Nacional Autonoma de Mexico, Mexico City, Mexico) was consulted, and the vegetal specimen was identified as *Salix babylonica* L. (IBUNAM: MEXU: 9744).

### 4.2. Preparation of the Hydroalcoholic Extract

The fresh leaves (4500 g) were washed and then dried at room temperature in the dark. The dried *Salix babylonica* leaves (2000 g) were macerated using a hydroalcoholic solution of water:ethanol (40:60 *v*/*v*)) at room temperature for 24 h to obtain an extract. The extract was filtered using Whatman filter paper (Whatman^®^ 42). After filtration, the solvent was eliminated using a rotary evaporator (Büchi R-300, Flawil, Switzerland, to obtain a semisolid extract, and this extract was lyophilized (LABCONCO^®^) and finally freeze-dried and stored at −4 °C, until the phytochemical analysis and antibacterial evaluation.

### 4.3. Identification of Major Compounds

The hydroalcoholic extract of the *Salix babylonica* leaves (SBHE) was processed for bipartition via liquid-liquid chromatography using water/ethyl acetate solvents (Merck, Darmstadt, Germany). Two fractions, an aqueous fraction (ASB) and an organic fraction (ACSB), were obtained. The solvent in both fractions was eliminated using low-pressure distillation (Büchi R-300, Flawil, Switzerland). Both fractions were evaluated through a pharmacological antibacterial test. The most active fraction, ACSB (13.2 g), was fractionated by chromatographic open column (20 × 600 mm), previously packed with silica gel 60 (Merck, mesh 70–230, 130 g). An n-hexane/EtOAC/MeOH gradient system was used as mobile phase, starting with 100% of hexane with successive increments of EtOAc. When the gradient system was 50:50, methanol was incorporated to the mobile phase, with increments of 10% MeOH until 100% (the volume of all samples was 250 mL). Twenty-nine fractions were obtained, which were grouped into 10 final fractions (F1AC to F10AC), according to their chemical composition. Each step of the chemical separation was monitored by thin layer chromatography (TLC) in a normal phase (silica gel at 60 F254, Merck, Darmstadt, Germany), and the plates were visualized under long UV (365 nm) and short UV lamps (254 nm) and were developed with chromogenic developers (cerium sulfate, 4-hydroxybenzaldehyde and polyethylene glycol, NP-PEG solutions). F8AC and F10AC were subjected to an HPLC method which revealed that these fractions contain the flavonoids: Luteolin and Luteoloside (Luteolin-7-*O*-glucoside), respectively. These flavones were identified by direct comparison by HPLC with analytical standards purchased from Sigma-Aldrich^®^.

The chemical composition of F7AC was analyzed on a Gas Chromatograph Agilent Technology 6890 (Wilmington, DE, USA), coupled with a 5973N mass detector, and the ionization mode was the Electronic Impact (IE) mode at 70 eV. Volatile compounds were separated on a HP 5MS capillary column (25 m long, 0.2 mm i.d., with a 0.3-µm film thickness). Oven temperature was set at 40 °C for 2 min, then programmed from 40‒260 °C at 10 °C/min and maintained for 20 min at 260 °C. The Mass detector conditions were as follows: interphase temperature, 200 °C, and mass acquisition range, 20-550. The injector and detector temperatures were set at 250 and 280 °C, respectively. The spitless injection mode was carried out with 1 µL of each fraction (3 mg/mL solution). The carrier gas was helium, at a flow rate of 1 mL/min. The identification of the compounds was conducted, comparing the mass spectra with those of the National Institute of Standards and Technology (NIST, 1.7 Library) and with data from the literature [36], this methodology allowed to preliminary identified five compounds (**3**–**7**)

### 4.4. Antibacterial Activity

To determinate the antibacterial activity, the Minimal Inhibitory Concentration (MIC) and Minimal Bactericidal concentration (MBC) were determined for the extract, fractions and subtraction from *Salix babylonica* against two Gram-positive bacteria (*Staphylococcus aureus*, ATCC^6538^, *Listeria monocytogenes,* ATTCC^19113^) and a Gram-negative bacterium (*Escherichia* coli, ATCC^35218^), in accordance with the CLSI guidelines [37].

#### 4.4.1. Minimal Inhibitory Concentration (MIC)

The broth micro-dilution method, described by Mothana et al., 2009, and Kaewpiboon et al., 2012, with modifications, was used to determine the Minimal Inhibitory Concentration (MIC) of the *Salix babylonica* hydroalcoholic extract [34,35]. Within sterile 96-well plates, two-fold serial dilutions of the *Salix babylonica* hydroalcoholic extract (concentration range 200 to 0.39 mg/mL) were prepared, in triplicate (100 μL well-1).

The bacterial cell suspension was adjusted to a 0.5 McFarland (Remel, R20421) standard (approximately 1.5 × 10^6^ Colony Corming Units (CFU) mL). Into each well, 10 μL was added. Kanamycin (AppliChem 4K10421™, Darmstadt, Germany) was used as a positive control (64 to 0.5 µg/mL), and nutrient broth (DIFCO ^®^), as a negative control. The plates were incubated at 37 °C for 24 h at 70 rpm.

After incubation, 20 μL of a 0.04% (*w*/*v*) p-iodonitrotetrazolium (Sigma-Aldrich 18377, St. Louis, MO, USA) solution was added to each well and incubated for 30 min. The MIC of the extract was determined from the lowest concentration, at which no growth of the microorganism was observed, as determined by the color change, from yellow to pink.

#### 4.4.2. Minimal Bactericidal Concentration (MBC)

After incubation and the previous addition of *p*-iodonitrotetrazolium, 5 μL from each well was inoculated in Mueller-Hinton agar (DIBICO ^®^) and incubated at 37 °C for 24 h. The MBC was the lowest concentration of antimicrobial agent that kills >99.9% of the initial bacterial population and where no visible growth of the bacteria was observed on the plates [5].

In order to determine if the evaluated compounds have bactericidal or bacteriostatic effects, the ratio of MBC/MIC was determined. The effect was considered bacteriostatic, when the ratio was greater than 4, and bactericidal, when the ratio was less than or equal to 4 [10].

### 4.5. Statistical Analysis

The MIC and MBC results were normalized using log10 and were analyzed by a completely randomized design through ANOVA using the general linear model (GLM). Differences among the means were assessed by Tukey’s multiple comparison statistical analysis at the *p* = 0.05 level of significance using the SAS program, version 9.0 [38].

## 5. Conclusions

Although there are studies on the antibacterial activity of *Salix babylonica* hydroalcoholic extract, the responsible compounds have not been identified. Some authors attributed this biological effect to the presence of flavonoids in the plant; however, this had not been clarified until now.

In this work, we evaluated the activity of hydroalcoholic extract, two fractions and ten sub-fractions of *Salix babylonica* against *Escherichia coli, Staphylococcus aureus,* and *Listeria monocytogenes*, a bioguided chromatographic separation of the organic fraction (ACSB) allowed us to identify 3′,4′,5,7-tetrahydroxyflavone (luteolin) and 3′,4′,5,7-tetrahydroxyflavone-7-*O*-glucoside (luteoloside) as the compounds responsible for the antibacterial activity. Furthermore, our study could be considered the first document the anti-listerial activity of *Salix babylonica* leaves and is the first report of the presence luteolin and luteoloside in this plant. The results suggest that luteolin and luteoloside could be an alternative for the treatment of diseases caused some bacteria, which affect the animal and human health.

## Figures and Tables

**Table 1:** Minimal Inhibitory Concentration of the *Salix babylonica* hydroalcoholic extract and fractions against *Escherichia coli, Staphylococcus aureus*, and *Listeria monocytogenes.*

Treatment mg/mL	*Escherichia coli*	*Staphylococcus aureus*	*Listeria monocytogenes*
**SBHE**	100.00 ^a^	25.00 ^a^	50.00 ^a^
**ASB**	12.50 ^c^	25.00 ^a^	3.12 ^c^
**ACSB**	6.25 ^d^	6.25 ^b^	1.56 ^d^
**F1AC**	NA	NA	NA
**F2AC**	NA	NA	NA
**F3AC**	NA	NA	NA
**F4AC**	ND	ND	ND
**F5AC**	ND	ND	ND
**F6AC**	ND	ND	ND
**F7AC**	6.25 ^d^	1.56 ^c^	0.78 ^e^
**F8AC**	25.00 ^b^	0.39 ^d^	1.56 ^d^
**F9AC**	ND	ND	ND
**F10AC**	12.50 ^c^	6.25 ^b^	12.50 ^b^
**Kanamicyn µg/mL**	4.00	4.00	1.00
**Water**	NA	NA	NA
***p* value**	0.0001	0.0001	0.0001

SBHE = *Salix babylonica* hydroalcoholic extract. ASB = Aqueous fraction. ACSB = Organic fraction. F1AC to F10AC = subfractions obtained from ACSB. NA = No activity. ND = No determined due to solubility problems. Different literals in the column show significant differences (*p* ≤ 0.05) among the compounds evaluated.

**Table 2:** Minimal Bactericidal Concentration of the *Salix babylonica* hydroalcoholic extract and fractions against *Escherichia coli*, *Staphylococcus aureus*, and *Listeria monocytogenes*.

Treatment mg/mL	*Escherichia coli*	*Staphylococcus aureus*	*Listeria monocytogenes*
SBHE	200.00 ^a^	50.00 ^a^	100.00 ^a^
ASB	NA ^d^	NA ^f^	100.00 ^a^
ACSB	50.00 ^b^	12.50 ^c^	3.12 ^c^
F7AC	NA ^d^	6.25 ^d^	0.78 ^e^
F8AC	25.00 ^c^	0.78 ^e^	1.56 ^d^
F10AC	25.00 ^c^	25.00 ^b^	25.00 ^b^
Kanamicyn µg/mL	4.00	16.00	16.00
Water	NA	NA	NA
*p* value	0.0001	0.0001	0.0001

SBHE = *Salix babylonica* hydroalcoholic extract. ASB = Aqueous fraction. ACSB = Organic fraction. F7AC, F8AC, and F10AC = subfractions obtained from ACSB. NA = No activity. Different literals in the column show significant differences (*p* ≤ 0.05) among the compounds evaluated.

**Table 3:** Preliminary chemical composition by the GC-MS of F7AC fraction of *Salix babylonica* hydroalcoholic extract.

Order of Elution	Retention Time (min)	Molecular Weight (a.m.u.)	Compound	Amount (%)
3	8.04	116	1,2-cyclohexanediol	6.58
4	16.4	224	(*E*)-4-(4-hydroxy-2,2,6-trimetyl-7-oxabicyclo [4.1.0] heptan-1-yl)but-3-en-2-one	10.08
5	17.54	196	(*E*)-2-(2,2,6-trimethyl-7-oxabicyclo [4.1.0]heptan-1-yl) prop-1-en-1-ol	72.09
6	23.18	370	bis(2-ethylhexyl) adipate	2.42
7	32.41	412	Dehydrodiosgenin	0.67

## References

[1] Mellor D.J. (2016). Updating Animal Welfare Thinking: Moving beyond the “Five Freedoms” towards “A Life Worth Living”. Animals.

[2] Bayvel A. In the OIE animal welfare strategic initiative–Progress, priorities and prognosis. Proceedings of the Global Conference on animal welfare: An OIE initiative.

[3] MacGowan A., Macnaughton E. (2017). Antibiotic resistance. Medicine.

[4] Webster J. (1995). Animal Welfare: A Cool Eye Towards Eden.

[5] Balouiri M., Sadiki M., Ibnsouda S.K. (2016). Methods for in vitro evaluating antimicrobial activity: A review. J. Pharm. Anal..

[6] Mostafa A.A., Al-Askar A.A., Almaary K.S., Dawoud T.M., Sholkamy E.N., Bakri M.M. (2018). Antimicrobial activity of some plant extracts against bacterial strains causing food poisoning diseases. Saudi J. Biol. Sci..

[7] Wahab A.G., Sallam A., Elgaml A., Farid Lahloub M.S., Afif M. (2018). Antioxidant and antimicrobial activities of *Salix babylonica* extracts. World J. Pharm. Pharm. Sci..

[8] Wang T., Li Q., Bi K. (2018). Bioactive flavonoids in medicinal plants: Structure, activity and biological fate. Asian J. Pharm. Sci..

[9] Mishra M.P., Rath S., Swain S.S., Ghosh G., Das D., Padhy R.N. (2017). In vitro antibacterial activity of crude extracts of 9 selected medicinal plants against UTI causing MDR bacteria. JKSUS.

[10] Djihane B., Wafa N., Elkhamssa S., Pedro D.H.J., Maria A.E., Mohamed Mihoub Z. (2017). Chemical constituents of Helichrysum italicum (Roth) G. Don essential oil and their antimicrobial activity against Gram-positive and Gram-negative bacteria, filamentous fungi and Candida albicans. Saudi Pharm. J..

[11] Bélanger L., Garenaux A., Harel J., Boulianne M., Nadeau E., Dozois C.M. (2011). Escherichia coli from animal reservoirs as a potential source of human extraintestinal pathogenic *E. coli*. FEMS Immunol. Med. Microbiol..

[12] Peton V., Le Loir Y. (2014). Staphylococcus aureus in veterinary medicine. Infect. Genet. Evol..

[13] Dhama K., Karthik K., Tiwari R., Shabbir M.Z., Barbuddhe S., Malik S.V.S., Singh R.K. (2015). Listeriosis in animals, its public health significance (food-borne zoonosis) and advances in diagnosis and control: A comprehensive review. Vet. Quart..

[14] Sulaiman G.M., Hussien N.N., Marzoog T.R., Awad H.A. (2013). Phenolic content, antioxidant, antimicrobial and cytotoxic activities of ethanolic extract of Salix alba. Am. J. Biochem. Biotechnol..

[15] Valladares-Carranza B., Felipe-Pérez Y.E., Zamora-Espinosa J.L., Velázquez-Ordoñez V., Díaz-González B.A.E., Gutiérrez-Castillo A., Ortega-Santana C., Sánchez-Torres J.E., Zaragoza-Bastida A., Rivero-Pérez N., Papst M. (2019). Listeriosis: An Emerging Pathology in Sheep. Sheep Diseases: Signs, Symptoms and Prevention.

[16] Salem A.Z.M., Salem M.Z.M., Gonzalez-Ronquillo M., Camacho L.M., Cipriano M. (2011). Major chemical constituents of *Leucaena leucocephala* and *Salix babylonica* leaf extracts. J. Trop. Agric..

[17] Kim S., Lee S., Lee H., Ha J., Lee J., Choi Y., Oh H., Hong J., Yoon Y., Choi K.H. (2017). Evaluation on Antimicrobial Activity of Psoraleae semen Extract Controlling the Growth of Gram-Positive Bacteria. Korean J. Food Sci. Anim. Resour..

[18] Whitehead S.R., Bowers M.D., McArthur C. (2014). Chemical ecology of fruit defence: Synergistic and antagonistic interactions among amides from Piper. Funct. Ecol..

[19] Popova T.P., Kaleva M.D. (2015). Antimicrobial Effect in vitro of Aqueous Extracts of Leaves and Branches of Willow (*Salix babylonica* L.). Int. J. Curr. Microbiol. App. Sci..

[20] Borges A., Ferreira C., Saavedra M.J., Simoes M. (2013). Antibacterial activity and mode of action of ferulic and gallic acids against pathogenic bacteria. Microb. Drug Resist..

[21] Ndhlala A.R., Ghebrehiwot H.M., Ncube B., Aremu A.O., Gruz J., Subrtova M., Dolezal K., du Plooy C.P., Abdelgadir H.A., Van Staden J. (2015). Antimicrobial, Anthelmintic Activities and Characterisation of Functional Phenolic Acids of Achyranthes aspera Linn.: A Medicinal Plant Used for the Treatment of Wounds and Ringworm in East Africa. Front. Pharmacol..

[22] Kaye K.S., Fraimow H.S., Abrutyn E. (2000). Pathogens resistant to antimicrobial agents: Epidemiology, Molecular Mechanisms, and Clinical Management. Infect. Dis. Clin. N. Am..

[23] Lin Y., Shi R., Wang X., Shen H.M. (2008). Luteolin, a flavonoid with potential for cancer prevention and therapy. Curr. Cancer Drug Targets.

[24] Seelinger G., Merfort I., Wölfle U., Schempp C.M. (2008). Anti-carcinogenic effects of the flavonoid luteolin. Molecules.

[25] Wittemer S.M., Ploch M., Windeck T., Müller S.C., Drewelow B., Derendorf H., Veit M. (2005). Bioavailability and pharmacokinetics of caffeoylquinic acids and flavonoids after oral administration of Artichoke leaf extracts in humans. Phytomedicine.

[26] Khodaie L., Delazar A., Nazemiyeh H. (2019). Biological Activities and Phytochemical Study of Pedicularis wilhelmsiana Fisch Ex. From Iran. Iran J. Pharm. Res..

[27] Tian C., Zhang P., Yang C., Gao X., Wang H., Guo Y., Liu M. (2018). Extraction process, component analysis, and in vitro antioxidant, antibacterial, and anti-inflammatory activities of total flavonoid extracts from abutilon theophrasti medic. leaves. Mediat. Inflamm..

[28] Khazaeli P., Mehrabani M., Mosadegh A., Bios S., Zareshahi R., Moshafi M.H. (2019). Identification of Luteolin in Henna (*Lawsonia inermis*) Oil, a Persion Medicine Product, by HPTLC and Evaluating Its Antimicrobial Effects. Res. J. Pharmacogn..

[29] Xiong J., Li S., Wang W., Hong Y., Tang K., Luo Q. (2013). Screening and identification of the antibacterial bioactive compounds from *Lonicera japonica* Thunb. Leaves. Food Chem..

[30] Wu T., He M., Zang X., Zhou Y., Qiu T., Pan S., Xu X. (2013). A structure–activity relationship study of flavonoids as inhibitors of *E. coli* by membrane interaction effect. Biochim. Biophys. Acta Biomembr..

[31] Ansari M., Emami S. (2016). β-Ionone and its analogs as promising anticancer agents. Eur. J. Med. Chem..

[32] Chunsriimyatav G., Dumaa M., Regdel D., Gerelt-Od Y., Selenge D. (2013). GC-MS analysis of some bioactive volatile constituents from aerial parts of *Polygonatum odoratum* (Mill. Druce). Int. J. Curr. Sci..

[33] Sharma V., Singh G., Kaur H., Saxena A.K., Ishar M.P.S. (2012). Synthesis of β-ionone derived chalcones as potent antimicrobial agents. Bioorg. Med. Chem. Lett..

[34] Mothana R.A., Lindequist U., Gruenert R., Bednarski P.J. (2009). Studies of the in vitro anticancer, antimicrobial and antioxidant potentials of selected Yemeni medicinal plants from the island Soqotra. BMC Complement. Altern. Med..

[35] Kaewpiboon C., Lirdprapamongkol K., Srisomsap C., Winayanuwattikun P., Yongvanich T., Puwaprisirisan P., Svasti J., Assavalapsakul W. (2012). Studies of the *in vitro* cytotoxic, antioxidant, lipase inhibitory and antimicrobial activities of selected Thai medicinal plants. BMC Complement. Altern. Med..

[36] Adams R.P. (2007). Identification of Essencial Oils Components by Gas Chromatography/Mass Spectometry.

[37] Clinical and Laboratory Standards Institute (2012). Methods for Dilution Antimicrobial Susceptibility Tests for Bacteria that Grow Aerobically (Approved Standards).

[38] SAS Institute (2006). SAS User’s Guide: Statistics.

